# Renal Dysfunction among Ghanaians Living with Clinically Diagnosed Hypertension in the Asutifi-South District: A Cross-Sectional Descriptive Study at the St. Elizabeth Hospital, Hwidiem

**DOI:** 10.1155/2018/8428063

**Published:** 2018-11-05

**Authors:** Sylvester Yao Lokpo, James Osei-Yeboah, William K. B. A. Owiredu, Francis Abeku Ussher, Verner Ndudiri Orish, Felix Gadzeto, Paul Ntiamoah, Felix Botchway, Ivan Muanah, Romeo Asumbasiya Aduko

**Affiliations:** ^1^Department of Medical Laboratory Sciences, School of Allied Health Sciences, University of Health and Allied Sciences, Ho, Ghana; ^2^Department of Molecular Medicine, School of Medical Sciences, Kwame Nkrumah University of Science and Technology, Kumasi, Ghana; ^3^Department of Clinical Biochemistry, Diagnostic Directorate, Komfo Anokye Teaching Hospital, Kumasi, Ghana; ^4^Faculty of Health and Allied Sciences, Koforidua Technical University, Koforidua, Eastern Region, Ghana; ^5^Department of Microbiology and Immunology, School of Medicine, University of Health and Allied Sciences, Ho, Ghana; ^6^Laboratory Department, St. Elizabeth Hospital, Catholic Health Services, Hwidiem, Brong Ahafo Region, Ghana; ^7^Department of Chemical Pathology, University of Ghana, Accra, Ghana; ^8^St. Elizabeth Hospital, Catholic Health Services, Hwidiem, Brong Ahafo Region, Ghana

## Abstract

**Background:**

This study aimed at evaluating the burden of renal dysfunction among people living with hypertension in the Asutifi-South District of the Brong Ahafo Region, who were attending clinic at the St. Elizabeth Hospital in Hwidiem.

**Methodology:**

A hospital-based, cross-sectional study was conducted among two hundred (200) hypertensive clients aged between 27 and 88 years who reported for clinical management from January to March, 2018. Data on sociodemography, comorbid disease status, antihypertensive medication, and their duration was obtained using a semistructured questionnaire and patient folders. Blood pressure, weight, and creatinine were measured using standard methods. Kidney function was assessed using Cockcroft Gault (CG), Four-Variable Modification of Diet in Renal Disease (4v-MDRD) and the Chronic Kidney Disease-Epidemiology Collaboration (CKD-EPI) equations. The 2012 Kidney Disease Improvement Global Outcome (KDIGO) Criteria were used to categorize renal function among study participants.

**Results:**

Renal impairment was observed among 25.00%, 9.50%, and 10.50% of study participants using CG, 4v-MDRD, and CKD-EPI equations, respectively. With the exception of CKD-EPI equation, females significantly recorded higher scores compared to their male counterparts (28.95% vs 12.5%, 11.84%, vs 2.08%) using CG and 4v-MDRD, respectively. Participants aged 50 years or more recorded the highest renal impairment.

**Conclusion:**

Renal dysfunction is common among people living with hypertension in the Asutifi-South District of the Brong Ahafo Region. Femininity, older age, disease comorbidity with diabetes, Thiazide diuretic and AR Blocker usage, and increasing duration of medication accounted for higher kidney dysfunction. Regular screening and management are therefore recommended to avert progression to end-stage renal failure (ESRD).

## 1. Introduction

The global public health importance of renal dysfunction is increasing due to a host of reasons including an increasing number of patients progressing to end-stage renal disease (ESRD), high costs to public health systems, and its associated morbidity and mortality, particularly those associated with cardiovascular disease [1]. The prevalence of hypertension is on the rise in Sub-Saharan Africa where most people with the disease remain undiagnosed, untreated, or inadequately treated [2]. Available information on hypertension prevalence in Ghana indicates rates ranging from 7.5% to 25.4% [3, 4]. There exist a multidirectional relationship between increased blood pressure (BP) and kidney pathology. The kidneys participate in the development and perpetuation of essential hypertension [5]. On the other hand, hypertension of any aetiology can lead to renal impairment (benign or malignant nephrosclerosis) and increased BP accompanied by proteinuria is an important factor related to the progression of kidney dysfunction [6]. Elevated BP damages blood vessels within the kidney, impairing its ability to filter fluid and waste from the blood, leading to an increase of fluid volume in the blood thus causing an increase in BP [7]. Despite being a principal public health issue, there is no surveillance system capable of detecting renal dysfunction at any stage among the populace as pertained in other developing nations across the world [8]. Prior to this study, there was very little information in the literature about the burden of kidney dysfunction among Ghanaians living with hypertension in Hwidiem in the Asutifi-South District of the Brong-Ahafo Region. In view of this, there is an urgent need for identifying hypertensive individuals with less than optimum kidney function with the aim of enhancing management approaches to reversing the disorder. This study is therefore aimed at evaluating the burden of renal dysfunction among hypertensive patients seeking medical care at the St. Elizabeth Hospital in Hwidiem in the Asutifi-South District of the Brong-Ahafo Region, Ghana.

## 2. Materials and Methods

### 2.1. The Study Site

Asutifi-South District is one of the administrative districts in the Brong Ahafo Region, Ghana. The District was carved out from the then Asutifi District in July 2012 with Hwidiem as the district capital. The dominant vocation of the district is agriculture with 71.8% of households engaged in activities such as crop farming, animal rearing, and fish farming. St. Elizabeth hospital is a Catholic Health delivery facility in the Goaso Diocese. It is located in the Asutifi-South District in the Brong Ahafo Region of Ghana, providing healthcare to all its neighboring communities. It started as a Leprosy camp in 1955 and evolved over the years into a District Hospital. The hospital provides the following services: curative, preventive and promotive, rehabilitative, diagnostic, and special programs [9].

### 2.2. Study Design

A hospital-based, descriptive cross-sectional study was conducted between January and March 2018 at the Diabetes and Hypertension Clinic of St. Elizabeth Hospital in the Asutifi-South District of the Brong Ahafo Region.

### 2.3. Sample Population

The study population consisted of male and female hypertensive participants who were of consent age (18 years and above) in the Asutifi-South District. The hypertensive registrants were conveniently and purposively sampled at the Diabetes and Hypertensive Clinic of the hospital.

### 2.4. Sample Size

Using the average monthly attendance of hypertensive patients for three months (133), a total study population of 399 was generated for the three months study duration. Raosoft online sample size calculator (http://www.raosoft.com/samplesize.html) was used, and a recommended minimum sample of 197 participants was calculated at 95% confidence level, 5% margin of error, and a response distribution of 50%.

### 2.5. Study Data Collection

#### 2.5.1. Socio-Demographic Data Capture (Questionnaire)

A self-reported semistructured questionnaire was administered to obtain primary data from consenting adult clients. Sociodemographic information captured included age, gender, marital status, educational level, and occupation. Diabetes status and therapeutic variables including type of antihypertensive medication used and the duration on medication were ascertained using patient folders.

### 2.6. Blood Pressure and Weight Measurement

Blood pressure was measured in the nondominant arm using fully-automated blood pressure monitor (OMRON Healthcare, Intelli-Sense BP785, HEM-7222, USA) in sitting position after resting for 3–5 minutes. A single qualified nurse recorded the average of two consecutive blood pressure readings taken on different occasions. Weight of participants was measured in light clothing, without shoes, and standing upright using a digital weighing scale (Health O Meter, USA) to the nearest 0.1 kg.

### 2.7. Blood Sampling and Laboratory Analysis

Venous blood sample was drawn from the antecubital veins of the arm of which three (3) milliliters was dispensed into a vacutainer® serum separator tube using the closed vacutainer system. The sample in the serum separator tube was allowed to clot, centrifuged at 2500 revolutions per minute (rpm) for 5 minutes at room temperature to obtain serum. The serum was stored at -20°C at the St. Elizabeth Hospital until analysis. Serum samples were then transported on ice to the St. John of God Hospital Laboratory, Duayaw-Nkwanta where it was thawed and creatinine was measured on a Random Access Fully Automated Dirui CS – T240 Chemistry Analyzer, China. The methods used to assay creatinine were predetermined by the reagent manufacturer (Dirui Industries Co., Ltd, China). The quality of the results was ensured by running daily quality control checks and regular calibration of the instruments used.

### 2.8. Estimated Glomerular Filtration Rate (eGFR) Calculation

The eGFR was calculated from serum creatinine values using the following predictive equations:

(I) Cockroft-Gault (CG) [10]
  (1) Males=140−Age×Weight72×Serum  Creatininemg/dlFemales=140−Age×Weight72×Serum  Creatinine×0.85
(II) Four-Variable Modification of Diet in Renal Disease (4v-MDRD) [11]
  (2) Males=186×SCr−1.154×Age−0.203×1.212Females=186×SCr−1.154×Age−0.203×1.212×0.74
(III) Chronic Kidney Disease Epidemiology collaboration- CKD-EPI [12]; see Table 1.

### 2.9. Definition of Renal Impairment

The calculated GFR was used to classify study participants into various categories of renal function according to the Kidney Disease Improvement Global Outcome (KDIGO) Criteria [13]. Renal impairment was defined as eGFR < 60 mL/min/1.73 m^2^ consistent with categories G3, G4, and G5 as indicated in Table 2.

### 2.10. Statistical Analysis

Normality of all continuous variables was tested. Continuous variables were expressed as their mean ± standard deviation. Gender variation in the prevalence of kidney function was performed using unpaired t-tests, chi-square (*χ*2) tests, or Fisher exact tests where appropriate. A level of p<0.05 was considered as statistically significant for all analyses. IBM Statistical Package for Social Sciences (SPSS Inc, Chicago, USA) (http://www.spss.com) version 22.00 and GraphPad Prism version 6.01 GraphPad software, San Diego, California USA (http://www.graphpad.com), for windows were used for statistical analysis

### 2.11. Ethical Consideration

Ethical approval was obtained from the Research Ethics Committee of the University of Health and Allied Sciences, Ho, Ghana** (UHAS-REC/A.5 **[35]** 17-18),** as well as written approval from management of St. Elizabeth hospital, Hwidiem. Informed consent from all participants was obtained following explanations and clarification of the purpose of the study. Data obtained from participants was kept confidential.

## 3. Results

Out of the two hundred (200) participants recruited into this study, 48 (24.00%) were males and 152 (76.00%) were females. The average age of the total population was 61 ± 10 years. Sixty-seven (33.50%) of the study participants were both diabetic and hypertensive while 133 (66.50%) were hypertensive only. Majority of the study population were married [127 (63.5%)], with only 22.00% attaining secondary level education or higher at the time of this study. Majority of the study participants were gainfully employed 161 (80.50%). The average weight of the study participants was 64.23 ± 13.2 kg with the difference in weight between participants presenting with both diabetes and hypertension and those suffering from only hypertension observed to be statistically comparable (see Table 3).

The prevalence of renal impairment ranged from 9.50% through 10.50 to 25.00% using the 4v-MDRD, CKD-EPI and CG equations respectively. About 50 (22.50%), 19 (8.00%), and 21 (8.50%) of study participants were found to present with mild to severely decreased GFR (G3) using CG, 4v-MDRD, and CKD-EPI, respectively, with less than 3% classified as having severely decreased GFR (G4) while none presented with kidney failure (G5). The prevalence of renal impairment was significantly tilted toward the diabetic and hypertensive group using the CG, 4v-MDRD, and CKD-EPI equations (see Table 4).

Significant gender variation in renal dysfunction was observed, with a higher prevalence tilted toward the female gender using the CG and 4v-MDRD equations except for CKD-EPI equation where the difference in the percentage scores was statistically comparable. The rate of renal impairment among the male participants ranged from 2.08% to 12.50% as compared to 11.84% to 28.95% among their female counterparts. All the male participants presenting with renal impairment were in G3 category (see Table 5).

Using the predictive equations, renal dysfunction was found to be highest in participants aged 70 years or more except for the 4v-MDRD and CKD-EPI equations where the scores were highest among the 50-59 years age group. Among the hypertensive medications, patients on Diuretics and AR Blockers presented with the highest scores using the predictive CKD equations. Generally, study participants on combination therapy of three medications presented with the greatest percentage of kidney dysfunction (see Table 6). Among the respondents presenting with renal impairment, there was a significantly increasing number of respondents from the first quartile through to the fourth quartile of antihypertensive medication duration whereas the reverse was observed among participants without evidence of renal damage (p=0.0301).

## 4. Discussion

In the present study, the prevalence of renal impairment among the study population was estimated at 25.00%, 9.50%, and 10.50% using CG, 4v-MDRD, and CKD-EPI predictive equations, respectively. With the exception of CG equation (29.85% vs 22.56%; p=0.3002), the prevalence of renal impairment among participants presenting with both diabetes and hypertension was significantly higher compared to participants presenting with only hypertension (Table 4). Ephraim and colleagues recorded a 30% prevalence of kidney dysfunction using the CKD-EPI definitive criteria among a high-risk population in the Sekondi-Takoradi Metropolis in South-Western Ghana [14]. A similar investigation conducted among hypertensive patients in Donkorkrom in the Eastern Region yielded a prevalence of 50%, 43%, and 46% using CG, MDRD, and CKD-EPI equations, respectively [15]. In other jurisdictions, a high burden of renal dysfunction among hypertensive individuals was reported in Nigeria [16, 17], Cameroon [18, 19], Guinea [20], Brazil [21], and Qatar [22]. However, renal dysfunction prevalence rates lower than the findings of this study have been reported among some Ghanaian (4.1% and 0.5%) [23] and South African (7.8%) [24] populations.

Although information is dearth in the present study to explaining the difference in kidney disease burden between the current work and others in various jurisdictions, a host of factors have been suggested to contribute to this phenomenon including difference in population characteristics, geographical location, ethnicity, hereditary, type of definitive criteria used in estimating kidney dysfunction, laboratory method employed and the presence of other CKD risk profiles as well as disease severity [14, 23, 24].

Gender preponderance to impaired renal function has been shown to be skewed toward the female gender [19]. In the present study, a significant proportion of female respondents recorded higher percentage scores compared to their male counterparts using CG and 4v-MDRD equations except for CKD-EPI equation where the difference in the renal dysfunction scores was statistically comparable (Table 5). Our results could be partly attributed to the greater health seeking behaviour among Ghanaian hypertensive females than males recorded in this study, notwithstanding the results compared with our previous findings, where femininity significantly accounted for renal dysfunction among a hypertensive population in the Kumasi Metropolis [23]. Though the relationship which exists between the female gender and kidney dysfunction in hypertension is not fully understood, it is suggested that obesity associated with femininity may play an indirect role in mediating the pathophysiological process [25]. The proposed pathway linking obesity to kidney dysfunction in females includes glomerular hyperfiltration with resultant albuminuria and eventual segmental glomerulosclerosis [19].

In the present study, using the predictive CKD equations, renal dysfunction was found to be highest in participants aged 70 years or more except for 4v-MDRD and CKD-EPI equations where the CKD scores were highest among the 50-59 years age group (Table 6). This suggests that increasing age is associated with a decline in renal function of participants. Age is widely documented as an independent risk factor for both hypertension and kidney dysfunction in the Ghanaian and other populations [14, 23, 26–28]. In advancing age, a fall in GFR is probably due to reductions in the glomerular capillary plasma flow rate, glomerular capillary ultrafiltration coefficient, afferent arteriolar resistance and increased glomerular capillary hydraulic pressure; the haemodynamic changes occur with structural alterations, including loss of renal mass; hyalinization of afferent arterioles and in some cases, development of efferent glomerular arterioles; increase in the percentage of sclerotic glomeruli and tubulointerstitial fibrosis [17, 29].

The advent of the Seventh Report of the Joint National Committee on Prevention, Detection, Evaluation, and Treatment of High Blood Pressure (JNC VII) 2003 guidelines has seen a surge in the treatment and control of hypertension with antihypertensive medications in people with kidney dysfunction [30]. However, there are recent reports indicating a probable role of antihypertensive medications in worsening kidney function [31, 32]. Among the antihypertensive medications used in this study, participants on Angiotensin II Receptor Blockers (ARB) (25.00%) and Thiazide Diuretics (15.38%) presented with the highest percentage scores using CG, 4v-MDRD, and CKD-EPI equations, respectively. Generally, study respondents on three combination therapy demonstrated greater percentage scores (Table 6). A significantly increasing number of respondents presenting with kidney dysfunction clustered at increasing quartile of duration of antihypertensive medication use (p=0.0301). [33]. Hawkins and Houston [34] observed a positive correlation between changes in the use of diuretics and increase in the occurrence of ESRD with a time lag of two years. Increased advanced stages of renal insufficiency, particularly among those with albuminuria after an increase in ARB polytherapy, have been reported [30].

The present study is limited in its cross-sectional design; hence causal relationship of renal impairment could not be established. Serum creatinine assay (picric acid method) adopted from the manufacturer was not traceable to the standardized isotope dilution mass spectrometry (IDMS). The study also relied on a single measurement of serum creatinine and estimation of GFR instead of two measurements three months apart in addition to urine protein estimation to determine urine albumin/creatinine ratio (ACR). This could lead to many missed cases of kidney damage.

## 5. Conclusion

The burden of renal dysfunction is high among Ghanaian hypertensive clients in the Asutifi-South District of the Brong-Ahafo Region. Females and participants presenting with comorbidity (both hypertension and diabetes) were most affected by renal dysfunction. Older age, thiazide diuretic and AR Blocker medications, increasing duration of antihypertensive therapy, and their combination were associated with greater kidney dysfunction. Regular screening and management are therefore recommended to avert progression to end-stage renal disease (ESRD).

## Figures and Tables

**Table 1:** 

**Gender**	**Serum Creatinine **µ**mol/L (mg/dL)**	**Estimated Glomerular Equation**
Female	≤62 (≤0.7)	eGFR=166×Serum Creatinine0.7-0.329×0.993Age
Female	>62 (>0.7)	eGFR=166×Serum Creatinine0.7-1.209×0.993Age

Male	≤80 (≤0.9)	eGFR=163×Serum Creatinine0.9-0.411×0.993Age
Male	>80 (>0.9)	eGFR=163×Serum Creatinine0.9-1.209×0.993Age

**Table 2:** 

**GFR Category**	**Description**	**Range **(ml/min/1.73 m^2^)
G1	Normal or high	≥90
G2	Mildly decreased	60 to 89
G3	Mildly to moderately decreased	30 to 59
G4	Severely decreased	15 to 29
G5	Kidney failure	<15

**Table 3:** Socio-demographic characteristic of the population under study stratified by disease status.

Parameter	Total	Diabetic and Hypertensive all together	Hypertensive only
Total Respondents	200 (100)	67 (33.5)	133 (66.5)
Age (years)	61 ± 10	62 ± 9	61 ± 10
Weight (kg)	64.23 ± 13.2	63.10 ± 13.8	64.80 ± 13.8
**Gender**			
Male	48 (24.0)	12 (17.9)	36 (27.1)
Female	152 (76.0)	55 (82.1)	97 (72.9)
**Marital Status**			
Single	3 (1.5)	1 (1.5)	2 (1.5)
Married	127 (63.5)	36 (53.7)	91 (68.4)
Divorced	9 (4.5)	5 (7.5)	4 (3.0)
Widowed	58 (29.0)	24 (35.8)	34 (25.6)
Separated	3 (1.5)	1 (1.5)	2 (1.5)
**Educational background**			
None	86 (43.0)	33 (49.3)	53 (39.8)
Basic	70 (35.0)	21 (31.3)	49 (36.8)
Secondary	21 (10.5)	9 (13.4)	12 (9.0)
Tertiary	23 (11.5)	4 (6.0)	19 (14.3)
**Employment Status**			
Unemployed	36 (18.0)	12 (17.9)	24 (18.0)
Employed	161 (80.5)	54 (80.6)	107 (80.5)
On Pension	3 (1.5)	1 (1.5)	2 (1.5)

Data is presented as mean ± standard deviation and frequency with percentage in parenthesis.

**Table 4:** Renal dysfunction categorization among study population using KDIGO eGFR Criteria stratified by disease status.

	eGFR Category		Total	Diabetic and Hypertensive all together	Hypertensive only	p-value
CG	G 1	(≥90)	73 (36.50)	22 (32.84)	51 (38.35)	
	G 2	(60 to 89)	77 (38.50)	25 (37.31)	52 (39.10)	
	G 3	(30 to 59)	45 (22.50)	17 (25.37)	28 (21.05)	
	G 4	(15 to 29)	5 (2.50)	3 (4.48)	2 (1.50)	
	G 5	(<15)	0 (0.00)	0 (0.00)	0 (0.00)	
	**Renal impairment (3+4+5)**		**50 (25.00)**	**20 (29.85)**	**30 (22.56)**	**0.3002**

4v-MDRD	G 1	(≥90)	137 (68.50)	41 (61.19)	96 (72.18)	
	G 2	(60 to 89)	44 (22.00)	15 (22.39)	29 (21.80)	
	G3	(30 to 59)	16 (8.00)	9 (13.43)	7 (5.26)	
	G4	(15 to 29)	3 (1.50)	2 (2.99)	1 (0.75)	
	G5	(<15)	0 (0.00)	0 (0.00)	0 (0.00)	
	**Renal impairment (3+4+5)**		**19 (9.50)**	**11 (16.42)**	**8 (6.02)**	**0.0227**

CKD-EPI	G1	(≥90)	130 (65.00)	40 (59.70)	90 (67.67)	
	G2	(60 to 89)	49 (24.50)	16 (23.88)	33 (24.81)	
	G3	(30 to 59)	17 (8.50)	8 (11.94)	9 (6.77)	
	G4	(15 to 29)	4 (2.00)	3 (4.48)	1 (0.75)	
	G5	(<15)	0 (0.00)	0 (0.00)	0 (0.00)	
	**Renal impairment (3+4+5)**		**21 (10.50)**	**11 (16.42)**	**10 (7.52)**	**0.00845**

Data is presented as frequency and percentage in parenthesis. 4v-MDRD – four variable Modification of Diet in Renal Disease, CG – Cockcroft-Gault, CKD-EPI – Chronic Kidney Disease Epidemiology collaboration, eGFR – estimated Glomerular Filtration Rate, CKD – Chronic Kidney Disease, G1-Category one, G2-Category two, G3-Category three, G4-Category four, G5-Category five.

**Table 5:** Renal dysfunction categorization among study population using KDIGO eGFR Criteria stratified by gender.

	eGFR Category		Male	Female	p-value
CG	G1	(≥90)	22 (45.83)	51 (33.55)	
	G2	(60 to 89)	20 (41.67)	57 (37.50)	
	G3	(30 to 59)	6 (12.50)	39 (25.66)	
	G4	(15 to 29)	0 (0.00)	5 (3.29)	
	G5	(<15)	0 (0.00)	0 (0.00)	
	**Renal impairment (3+4+5)**		**6 (12.50)**	**44 (28.95)**	**0.0222**

4v-MDRD	G1	(≥90)	38 (79.17)	99 (65.13)	
	G2	(60 to 89)	9 (18.75)	35 (23.03)	
	G3	(30 to 59)	1 (2.08)	15 (9.87)	
	G4	(15 to 29)	0 (0.00)	3 (1.97)	
	G5	(<15)	0 (0.00)	0 (0.00)	
	**Renal impairment (3+4+5)**		**1 (2.08)**	**18 (11.84)**	**0.0487**

CKD-EPI	G1	(≥90)	37 (77.08)	93 (61.8)	
	G2	(60 to 89)	9 (18.75)	40 (26.32)	
	G3	(30 to 59)	2 (4.17)	15 (9.87)	
	G4	(15 to 29)	0 (0.00)	4 (2.63)	
	G5	(<15)	0 (0.00)	0 (0.00)	
	**Renal impairment (3+4+5)**		**2 (4.17)**	**19 (12.50)**	**0.1136**

Data is presented as frequency with percentage in parenthesis. 4v-MDRD: four-variable modification of diet in renal disease, CG: Cockcroft-Gault, CKD-EPI: chronic kidney disease epidemiology collaboration, eGFR: estimated Glomerular Filtration Rate, CKD: chronic kidney disease, G1: category one, G2: category two, G3: category three, G4: category four, and G5: category five.

**Table 6:** Prevalence of renal dysfunction stratified by age and medication.

**Parameter**	**CG**	**Rank**	**4v-MDRD**	**Rank**	**CKD-EPI**	**Rank**
**Age Category (years)**						
<50	2 (7.40)	4th	1 (3.70)	4th	1 (3.70)	4th
50-59	11 (22.00)	2nd	10 (20.00)	1st	10 (20.00)	1st
60-69	19 (21.35)	3rd	5 (5.62)	3rd	6 (6.74)	3rd
≥70	18 (56.25)	1st	3 (9.38)	2nd	4 (11.76)	2nd
**Antihypertensive Medication**						
ACE Inhibitor	27 (24.32)	2nd	10 (9.01)	2rd	11 (9.91)	3rd
CC Blocker	36 (20.34)	4th	14 (7.91)	3rd	18 (10.17)	2nd
Diuretics	6 (23.08)	3rd	4 (15.38)	1st	4 (15.38)	1st
AR Blocker	17 (25.00)	1st	6 (8.82)	4th	6 (8.82)	4th
*β*-Blocker	1 (12.50)	5th	0 (0.00)	5th	0 (0.00)	5th
One	7 (29.17)	3rd	2 (8.33)	3rd	3 (12.50)	2nd
Two	24 (25.00)	4th	9 (9.38)	2nd	8 (6.56)	4th
Three	6 (31.58)	2nd	3 (15.79)	1st	8 (18.18)	1st
Four	1 (33.33)	1st	0 (0.0)	4rh	1 (10.00)	3rd

Data is presented as frequency with percentage in parenthesis. 4v-MDRD: four-variable modification of diet in renal disease, CG: Cockcroft-Gault, CKD-EPI: chronic kidney disease epidemiology collaboration. ACE inhibitor: angiotensin converting enzyme inhibitor, and CC blocker: calcium channel blocker.

## Data Availability

The data used to support the findings of this study are available from the corresponding author upon request.
